# Enhancement of visual attention precedes the emergence of novel metaphor interpretations

**DOI:** 10.3389/fpsyg.2015.00892

**Published:** 2015-06-26

**Authors:** Asuka Terai, Masanori Nakagawa, Takashi Kusumi, Yasuharu Koike, Koji Jimura

**Affiliations:** ^1^Department of Human System Science, Graduate School of Decision Science and Technology, Tokyo Institute of TechnologyTokyo, Japan; ^2^Division of Cognitive Psychology in Education, Graduate School of Education, Kyoto UniversityKyoto, Japan; ^3^Precision and Intelligence Laboratory, Tokyo Institute of TechnologyTokyo, Japan; ^4^Department of Biosciences and Informatics, Keio UniversityYokohama, Japan

**Keywords:** creativity, metaphor comprehension, free spoken response, semantic attention, conceptual combination

## Abstract

Emergence of creative ideas often involves sudden linking of unrelated ideas. Here we demonstrate that the production of insights about novel metaphor was preceded by prolonged visual inspection of metaphorical sentences. The participants made free spoken responses in a task requiring the generation of semantic interpretations about visually presented sentences, where gaze locations were continuously monitored. We found that creative interpretations were primarily generated from novel metaphorical expressions, rather than from conventional expressions. Moreover, presented words within novel metaphors were visually inspected for longer periods specifically before creative interpretations were produced. Interestingly, prolonged gazes occurred several seconds (∼8 s) prior to the generation of creative interpretations, particularly, in the case of the topic word within the metaphor. These results demonstrate latent cognitive process meditating the emergence of insights, and suggest that the prior visual inspection prompted the semantic representation of the metaphorical sentences, which eventually facilitates creative ideas.

## Introduction

Creation of novel ideas involves combining of separate concepts that occurs infrequently and unexpectedly (Turner and Fauconnier, 1999). Creative ideas emerge from the convergence of multiple concepts, with creativity being enhanced by the distinctiveness of the concepts (Nagai et al., 2009). On the other hand, the emergence of novel ideas is primed by signature processes, such as sudden reorganizations of mental representation (Bowers et al., 1990; Seifert et al., 1994). However, reflecting the unexpected nature of the emergence, little is known about the temporally evolving processes underlying the generation of creative ideas.

Metaphor is a rhetorical expression that compares one concept to another concept to express the first concept in a concise and impressive manner (Lakoff and Johnson, 1980). Previous theoretical works have postulated the comprehension of metaphors in terms of the attributions of the concepts [e.g., Class Inclusion Theory (Glucksberg and Keysar, 1990); Structure Alignment Theory (Gentner et al., 2001)]. Importantly, however, metaphor comprehension requires the combining of different concepts, which involves cognitive processes that are comparable to those involved in the creation of novel thoughts (Becker, 1997; Gineste et al., 2000). One notable phenomenon is the “emergence” of interpretations (Tourangeau and Rips, 1991), where an interpretation of a metaphor is not related to either of the original concepts, but a combination of the two concepts provides for a novel interpretation to emerge^1^. These characteristics of metaphor interpretation can provide a unique opportunity for examining the emergence of creative thoughts, and possibly uncovering an alternative mechanism for metaphor comprehension.

The present study asks what specific situations enhance creative interpretations, and what processes precede their emergence. Previous studies suggest that inspection of visually presented items reflects attentional shifts and plays critical roles for higher-level cognitive functions, such as decision-making (Glaholt and Reingold, 2011), problem solving (Knoblich et al., 2001), and sentence comprehension (Rayner, 1998). For example, during the reading of visually presented sentences, the gaze fixates longer on a word when extra semantic attention is needed (Ehrlich and Rayner, 1981; Frazier and Rayner, 1990), and spatial shifts in visual attention precede insights in problem solving (Knoblich et al., 2001). These empirical evidences suggest that tracking visual attention is potentially beneficial for examining the cognitive processes underlying the emergence of creative interpretations.

**FIGURE 1:**
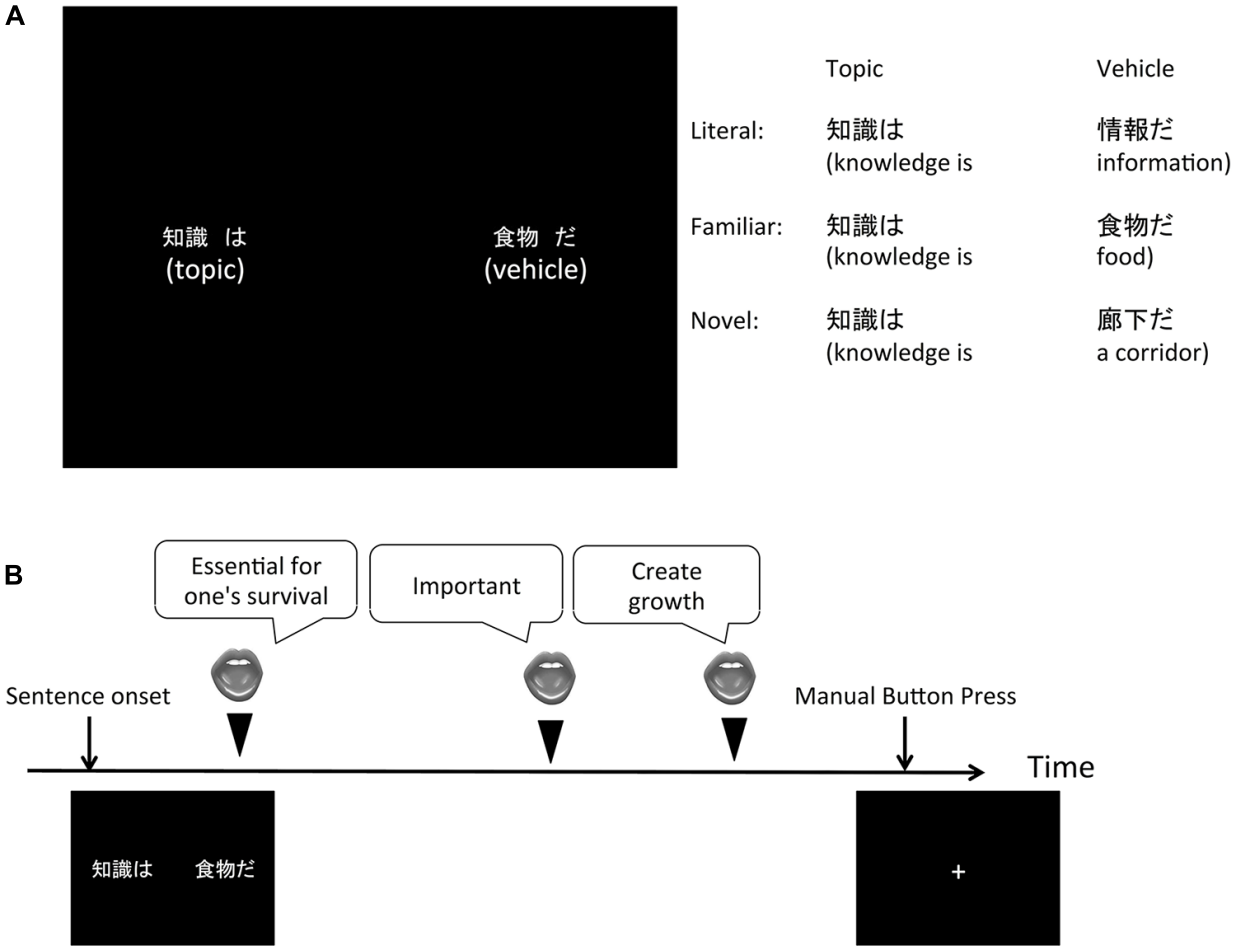
**Experimental design. (A)** Visual stimulus set during the present behavioral task. Participants were visually presented by sentences of the form “ ‘*topic’* is ‘*vehicle*’ ”, and instructed to think of interpretations of the sentences. **(B)** Temporal sequence of the task. Participants freely generated interpretations about the sentences and made spoken responses. They made a manual button press when they could not come up with more responses.

In order to address the issue above, the present study examines the temporal dynamics of visual attention while participants freely generated interpretation for visually presented metaphorical sentences (**Figure 1**). Responses were made vocally, and eye gaze was continuously monitored during the task. The sentences were manipulated such that they involved either semantically unrelated or related concept combinations (**Figure 1A**). We then examined the temporal patterns for gazes prior to the generation of creative interpretations.

## Materials and Methods

In order to address the issue above, the present study examines the temporal dynamics of visual attention while participants freely generated interpretation for visually presented metaphorical sentences (**Figure 1**). Responses were made vocally, and eye gaze was continuously monitored during the task. The sentences were manipulated such that they involved either semantically unrelated or related concept combinations (**Figure 1A**). We then examined the temporal patterns for gazes prior to the generation of creative interpretations.

### Participants

One-hundred twenty-seven undergraduate and graduate students participated in the study, and were native Japanese speakers. They consisted of a group of 25 participants [21 male and 4 female; mean age 20.1 (SD 1.68)], who participated in an interpretation generation experiment and another group of participants (*N* = 102) who participated in a separate experiment evaluating the responses and materials from the interpretation generation experiment. No participants participated in both experiments. The participants in the interpretation generation experiment were graduate and undergraduate students in Tokyo Institute of Technology and their educational backgrounds are engineering and natural science. They received 3,000 yen for their participation. The sample size of the interpretation generation experiment was pre-determined (∼25) based on the results of pilot sessions. The sample size of the evaluation experiment was also pre-determined (∼100) based on our prior experiences of questionnaire experiments in this domain. The data collection was stopped when sufficient samples were collected. The data in the pilot sessions were excluded in the analyses. Two participants from the interpretation generation experiment were discarded from the data analysis due to a low number of responses. All study protocols were approved by the Institutional Review Board of Tokyo Institute of Technology. The IRB approval did not allow us to collect personal information for the current secondary evaluation experiment.

### Interpretation Generation Experiment

#### Behavioral Procedure

Participants were tested individually in a testing room. They were seated facing a 17-inch LCD monitor located approximately 45 cm from the participants’ eyes. In order to accurately track eye movements without imposing any physical restrictions to jaw movements when making spoken responses, participants’ heads were stabilized by placing their foreheads to a plate.

The participants made free responses in an interpretation generation task about the stimuli sentences (e.g., Gentner and Clement, 1988; Becker, 1997; Gineste et al., 2000). In the present task, a sentence was visually presented on the screen, and participants were instructed to interpret it, and to make a free spoken response concerning their interpretation. The sentences involved metaphorical expressions of the form of “*A* is *B*”, (in Japanese, “*A* 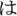 *B* 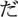
”), where *A* and *B* are referred to as the “*topic*” and “*vehicle*” words, respectively, (Richards, 1936; **Figure 1A**).

Three types of sentences were presented (see Supplementary Table 1): Novel metaphors (N), Familiar metaphors (F), and Literal sentences (L) which served as a control condition (e.g., Ahrens et al., 2007; Shibata et al., 2007a,b; Stringaris et al., 2007). The sentences for the F condition (30 sentences) were collected from previous studies of familiar metaphor interpretations (Nakamoto and Kusumi, 2004; Taira and Kusumi, 2008). The sentences and metaphors for the L and N conditions (30 sentences each) were created by replacing the vehicle words of the F sentences by another word, in order to control for the topic words across the conditions. For the N condition, the vehicle words included both concrete and abstract nouns pseudo-randomly collected from a corpus of newspaper articles (MAINICHI Newspaper, 1993–2002), such that the topic and vehicle words were conceptually and semantically unrelated. For the L condition, the vehicle words were chosen from definition sentences in a Japanese dictionary (Nihon-Daihyakka-Zensho, 2011, Shogakukan, Tokyo Japan) in order to maximize the degree of relatedness between the topic and vehicle words. The three conditions were pseudo-randomly presented.

The classification of these three conditions (N, F, and L) was evaluated in the other participant group (*N* = 102; see also below), where the semantic novelty of each sentence was rated on a 7-point scale (7: very novel; 1: very conventional). Significant differences in the novelty score were observed [N > F: *t*(101) = 6.5, *p* < 0.001, Holm corrected, *r* = 0.54; F > L: *t*(101) = 9.4, *p* < 0.001, corrected, *r* = 0.69], confirming that the conditions (N, F, and L) were appropriately distinct in terms of their metaphorical novelty. The entire set (30 novel metaphors, 30 familiar metaphors, and 30 literal sentences) was then divided into two subsets, with each involving 45 sentences (15 sentences from the N, F, and L conditions). Each participant was presented one of the two subsets.

In each trial, a sentence (“*A* 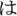 *B* 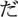
”) was presented on the screen after presentation of a fixation cross. The topic (A) and the vehicle (B) words were presented on the left and right sides of the screen, respectively, approximately 30° from the center (**Figure 1A**). The word “
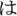
” was always presented immediately to the right to the topic word, and the word “
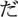
” was always presented immediately to the right of the vehicle word. The participants pressed a key on a keyboard when they could no longer come up with interpretation responses (**Figure 1B**). The sentence then disappeared, and was followed by the fixation cross for a period of 12 s. The fixation cross moved from a left, to a center, and then to a right location on the screen every 2–3 s, and the participants were instructed to follow the fixation cross with their eyes. This request sought to correct for shifts of eye positions due to head movements due to making spoken responses. The participants were allowed to take a short break after every five trials, and 45 trials were presented to each participant.

Spoken responses made during the experiment were recorded through an acoustic microphone. Image data for one eye was also recorded at a sampling rate of 60 Hz using a CCD camera sensitive to infrared rays, and monitored by the ViewPoint Eye Tracker (Arrington Research, Scottsdale, AZ, USA). Eye position was calibrated before the first run and recalibrated after every short break.

A post-task session was conducted on the same day in which the each participant rated the semantic unrelatedness between the responses that he/she generated him/herself during the trials and the topic/vehicle words presented in the trials. The ratings were on a 7-point scale (7: very unrelated; 1: very related). They also rated novelty of the presented sentences on a 7-point scale (7: very novel; 1: very conventional).

#### Analysis Procedure

Spoken recordings were analyzed oﬄine to determine response onsets (Asari et al., 2008). Each spoken responses was then classified as either a creative (emergent: E) or a conventional (non-emergent: NE) interpretation. This classification was based on the semantic unrelatedness rating score from the post-task session. More specifically, if the unrelatedness score was between 5 (relatively unrelated) and 7 (very unrelated), the response was classified as E, but otherwise, the response was classified as NE. This procedure sought to ensure that E responses were semantically unrelated to the topic and vehicle words, and that the interpretation emerged when the two words were combined within a single sentence. On the other hand, the NE interpretations can be attributed to the semantic characteristics of the topic and/or vehicle words.

During the task, the eye tracker calculated the “point of regard” (POR) on the basis of pupil position, which represents the time-series of *X*–*Y* coordinates with respect to the screen. Due to inevitable temporal fluctuations for PORs during and immediately before and after eye blinks, POR data points were discarded for the period from 100 ms prior to eyelid closures to 300 ms after closures (Asari et al., 2008). The POR time series were temporally smoothed with a 3-Hz Gaussian filter.

In order to determine which of the topic and vehicle words was been gazed at during interpretation generation, the POR data was analyzed along the *x*-axis. The time series for each trial was centralized and normalized based on the data taken during the fixation periods after each trial in order to minimize signal shift and drift between trials (as previously explained; -0.5: far left; 0: center; 0.5: far right).

We then calculated the cumulative gaze duration (CGD) for each topic and vehicle words prior to the generation response. CGD was defined as the duration of the gaze period temporally accumulated while the absolute POR along the *x*-axis exceeded 0.1, approximately corresponding to 15° from the screen center (vehicle and topic words were presented at *X* = 0.22 and -0.22, respectively). For each generated spoken response, accumulation started from response onset, and was temporally integrated backward until the point in time corresponding to the average of the overall duration of response (DR; see Results for more details). Note that, by definition, the maximum CGD is same as the average DR. In order to ensure stable gazes, periods with continuous gaze durations shorter than 0.1 s were discarded from the accumulation calculations.

### Evaluation Experiment for Responses and Sentences

In order to validate the *post hoc* classification of the responses (E and NE) according to the participants’ ratings of “unrelatedness” and the labeling of the sentences (N, F, L), a separate experiment was conducted. In the experiment of the study, another group of participants (*N* = 102) evaluated the degree of semantic unrelatedness between the spoken responses generated and the topic/vehicle words presented in the free interpretation task on a 7-point scale (7: very unrelated; 1: very related).

Spoken responses were first collected from all participants of the free interpretation task. Then, for each sentence trial, two E and two NE responses were pseudo-randomly sampled from individual participants; these represented 28.2 and 10.1% of the entire set of E and NE responses (667 kinds of E responses and 1941 kinds of NE responses), respectively. The sampled set was then divided into eight subsets with response types (E, NE) and sentence condition (N, F, L) matched within each. These procedures sought to reduce the total number of ratings for individual participants, and each subset involved 24 E and 24 NE responses. The participants evaluated one of the eight data sets, and each dataset was evaluated by 12 or 13 participants. Additionally, they rated the overall semantic novelty of the sentence presented in the free interpretation task on a 7-point scale (7: very novel; 1: very conventional). The participants marked their ratings on scales printed in a booklet.

### Statistical Analysis

Statistical analyses of behavioral data generated during the interpretation generation task were performed using two-way repeated measures ANOVA, with sentence conditions (N, F, and L) and response types (E, and NE) as factors. The unrelatedness ratings were also analyzed, using two-way repeated measures ANOVA. The novelty ratings were analyzed using one-way repeated measures ANOVA with sentence conditions (N, F, L) as the factor. *Post hoc t*-tests were also performed, and *p*-values were corrected based on Holm’s method.

## Results

**FIGURE 2:**
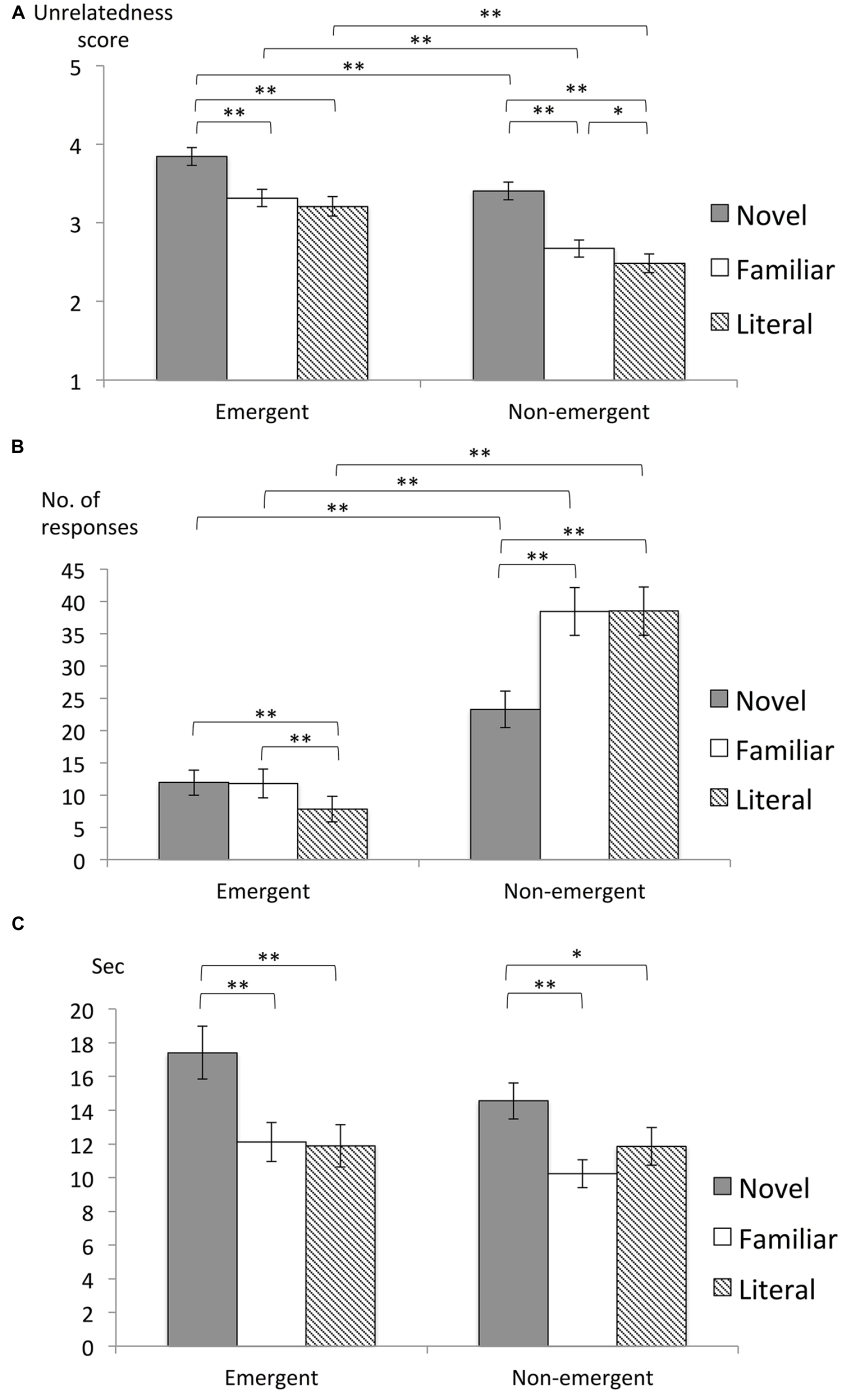
**(A)** Average ratings for the degree of semantic unrelatedness between generated responses and presented words (7: very unrelated; 1: very related) within the evaluations from the independent participant group (*N* = 102). **(B)** Average frequencies of responses in the free interpretation experiment. **(C)** Average durations to responses (DR) defined as the duration between the onsets of serial responses. Error bars represent the SEM across participants. ^∗^*p* < 0.05; ^∗∗^*p* < 0.01.

We first sought to verify the classifications of the E and NE responses by analyzing semantic unrelatedness ratings for the presented topic/vehicle words and the generated responses. Sampled responses (see Materials and Methods for details) were rated by an independent participant group (*N* = 102; see also Materials and Methods for details; **Figure 2A**). A repeated measures two-way ANOVA with response type (E, NE) and sentence (N, F, L) as factors revealed main effects of both response type [*F*(1,101) = 87.7, *p* < 0.001, $\eta_{p}^{2}$ = 0.46] and sentence [*F*(2,202) = 40.0, *p* < 0.001, $\eta_{p}^{2}$ = 0.28]. *Post hoc t*-tests further revealed that the unrelatedness scores for E responses were higher than those for the NE responses in all sentence conditions [E-N > NE-N: *t*(101) = 4.6, *p* < 0.01, corrected for multiple comparisons based on Holm’s method, *r* = 0.42; E-F > NE-F: *t*(101) = 7.5, *p* < 0.01, corrected, *r* = 0.59; E-L > NE-L: *t*(101) = 6.6, *p* < 0.01, corrected, *r* = 0.55]. The unrelatedness scores for interpretations of the N sentences were higher than those for both F and L sentences [N > F: *t*(101) = 7.3, *p* < 0.001, corrected, *r* = 0.59; N > L: *t*(101) = 6.7, *p* < 0.001, corrected, *r* = 0.56]. These results from the independent participant group validate the classifications of the E and NE responses and the subjective unrelatedness scores of the participants in the interpretation task (Tourangeau and Rips, 1991).

Next, we analyzed all the responses generated in the free interpretation experiment, and calculated the frequencies of responses (**Figure 2B**). A two-way repeated measures ANOVA was conducted with sentence conditions (N, F, L) and response types (E, NE) as factors. Significant main effects were observed for both sentences [*F*(2,44) = 24.3, *p* < 0.001, $\eta_{p}^{2}$ = 0.52] and response types [*F*(1,22) = 33.7, *p* < 0.001, $\eta_{p}^{2}$ = 0.61], as well as significant interaction [*F*(2,44) = 33.0, *p* < 0.001, $\eta_{p}^{2}$ = 0.60]. *Post hoc t*-tests further revealed that E responses were less frequently given than NE responses in all sentence conditions [NE-N > E-N: *t*(22) = 3.2, *p* < 0.01, corrected, *r* = 0.56, NE-F > E-F: *t*(22) = 5.9, *p* < 0.01, corrected, *r* = 0.78, NE-L > E-L: *t*(22) = 6.8, *p* < 0.01, corrected, *r* = 0.83]. Moreover, E responses were generated more frequently for the N and F sentences than for the L sentences [E-L < E-N: *t*(22) = 3.8, *p* < 0.01, corrected, *r* = 0.63; E-L < E-F: *t*(22) = 3.7, *p* < 0.01, corrected, *r* = 0.62]. These findings suggest that E responses were more infrequent, although they were mainly observed in the metaphorical (N, F) conditions. On the other hand, NE responses for N sentences were generated less frequently than such responses for the F and L sentences [NE-N < NE-F: *t*(22) = 7.8, *p* < 0.01, corrected, *r* = 0.87; NE-N < NE-L: *t*(22) = 5.9, *p* < 0.01, corrected, *r* = 0.78], suggesting that NE responses were generated more from conventional expressions (F, L) than from novel expressions (N), and, thus, that the N sentences were more challenging to interpret.

We then analyze temporal data for serial responses in order to examine the duration to generate interpretations. Durations to responses (DR) were defined as the interval between the onset of the response and the onset of the immediately prior response (regardless of response type). DRs thus reflect the time required to produce a response (**Figure 2C**). A two-way repeated measures ANOVA was conducted with response type (E, NE) and sentence condition (N, F, L) as factors. A significant main effect of sentence was observed [*F*(2,44) = 17.1, *p* < 0.001, $\eta_{p}^{2}$ = 0.44]. *Post hoc t*-tests further revealed that DRs were longer during N sentences than both L and F sentences [N > F: *t*(22) = 5.3, *p* < 0.01, corrected, *r* = 0.75; N > L: *t*(22) = 3.9, *p* < 0.01, corrected, *r* = 0.64], suggesting that N sentences required longer durations to produce interpretations, which is consistent with the lower frequency of responses for N sentences.

**FIGURE 3:**
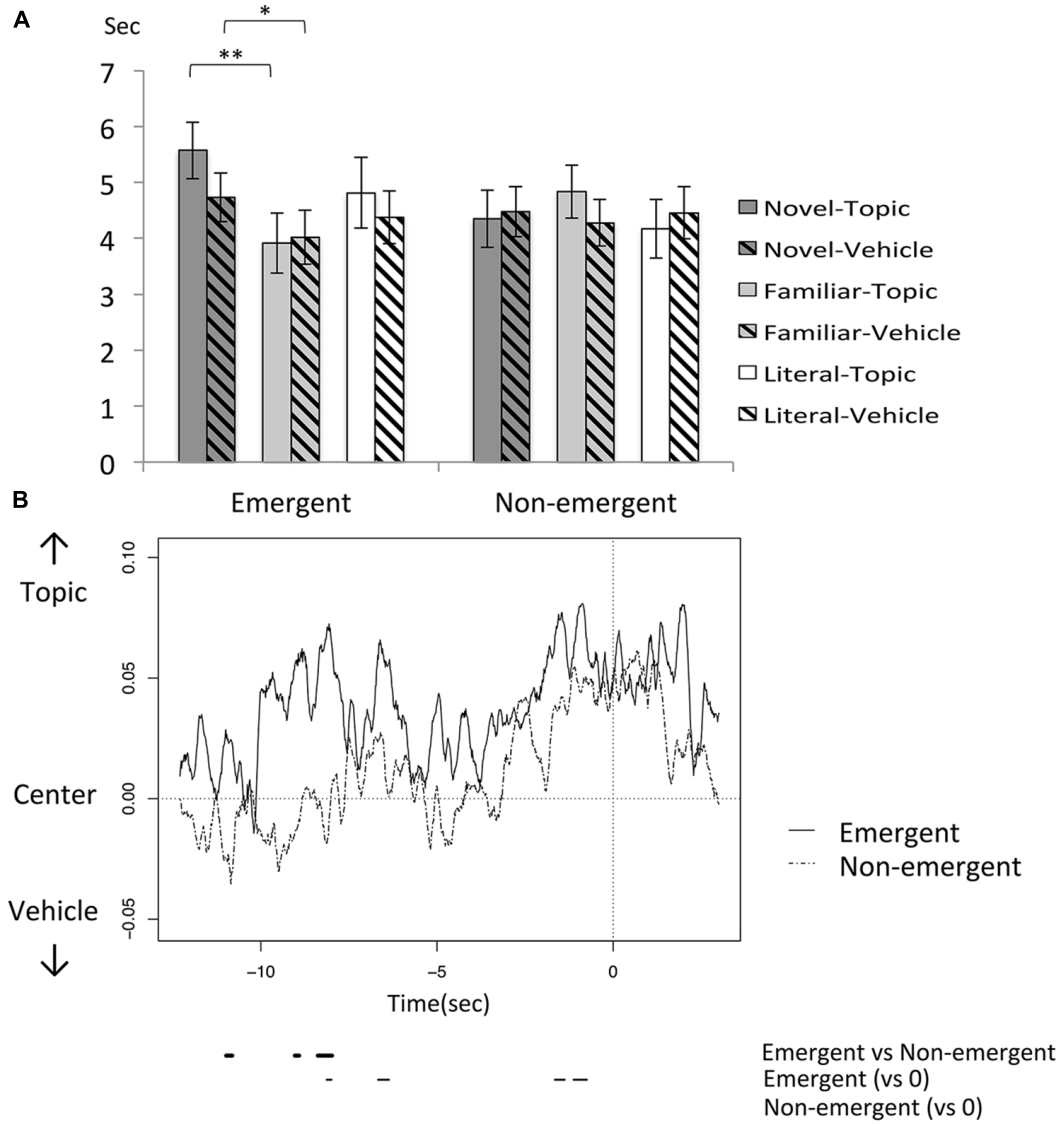
**(A)** Cumulative gaze durations (CGDs) to generate responses. Error bars represent SEM. ^∗^*p* < 0.05; ^∗∗^*p* < 0.01. **(B)** The time courses of *x*-axis gaze points within the N condition. The horizontal and vertical axes indicate time and normalized gaze points, respectively, and the time 0 indicates the onset of a spoken response. The solid lines (*t* < 0) below the time courses indicate the point when the gaze was shifted toward the topic and vehicle words and where the difference in gaze points (E vs. NE) was significant (*p* < 0.05).

Next, the eye tracking data was analyzed in order to examine visual attention toward the sentences. We first calculated the CGDs for both the topic and vehicle words during response generation, as the overall average DR period (12.3 s; see Materials and Methods for more details). As shown in **Figure 3A**, CGDs are assumed to reflect the total gaze durations when generating responses.

We first analyzed CGDs for topic words. A two-way repeated measures ANOVA for CGDs was conducted with response type (E, NE) and sentence condition (N, F, L) as factors. A significant main effect of sentence [*F*(2,44) = 3.4, *p* < 0.05, $\eta_{p}^{2}$ = 0.13] was observed, as well as an interaction between sentence and response type [*F*(2,44) = 5.5, *p* < 0.01, $\eta_{p}^{2}$ = 0.20]. *Post hoc t*-tests further revealed longer CGD for the N sentence than F sentence [N > F: *t*(22) = 2.9, *p* < 0.05, corrected, *r* = 0.53]. Most importantly, production of E responses within the N sentence condition were preceded by longer CGDs than within F sentence condition [E-N > E-F: *t*(22) = 3.4, *p* < 0.01, corrected, *r* = 0.58]. These results suggest that greater visual attention was given to the topic word during the generation of E responses within the N sentence, which is consistent with the suggestion that E responses were challenging to generate.

Next, for vehicle words, a similar two-way repeated measures ANOVA was conducted. A significant main effect of sentence was observed [*F*(2,44) = 11.9, *p* < 0.01, $\eta_{p}^{2}$ = 0.35], although the interaction effect failed to reach significance. *Post hoc t*-tests revealed longer CGDs for the N sentence condition [N > F: *t*(22) = 6.3, *p* < 0.01, corrected, *r* = 0.80, L > F: *t*(22) = 2.9, *p* < 0.05, corrected, *r* = 0.52], suggesting that interpretation generation within the N sentence conduction is associated with greater visual attention to vehicle words in addition to topic words.

**Figure 3B** presents the time courses of gaze point for both E and NE responses within N sentences. A sliding-window *t*-test (window size = 10 sample frames = 0.17 s) revealed significant gaze shifts to the topic word around -8, -7, and -2 s prior to E responses, but such shifts were not observed prior to NE responses. Accordingly, differences in gaze points were significant for E responses compared to NE responses at -11, -9, -8 s, shifting toward the topic words. These results suggest that the topic words received more visual attention during an earlier period in the generation of E responses, possibly reflecting the processes underlying the generation of creative interpretations.

## Discussion

Previous theoretical studies of metaphor comprehension have postulated that the interpretation of metaphors involves a conceptual interaction between topic and the vehicle representations (Black, 1962), and a combination of the topic and vehicle concepts brings about emergent interpretations (Gineste et al., 2000). Class Inclusion Theory (Glucksberg and Keysar, 1990) further extended this theoretical account by introducing a selective representation of vehicle characteristics that guides the attribution dimension related to the topic concept, and the theory was verified experimentally (Glucksberg et al., 1997; McGlone and Manfredi, 2001). It has also been hypothesized that the category representation of the vehicle is formed in an *ad hoc* manner (so-called *ad hoc* category), and then transformed into topic attribution during the generation of emergent interpretations (Vega Moreno, 2004). The present study provides experimental evidence for these hypotheses by demonstrating that sentence words received prolonged visual attention prior to the generation of emergent interpretations.

In novel metaphors, because the topic and vehicle words were semantically dissimilar, a broader semantic search is required to construct the *ad hoc* category for the vehicle information (Stringaris et al., 2007). This idea is compatible with the present findings that novel metaphors were associated with longer durations in the generation of interpretations. On the other hand, the enhanced visual attention prior to emergent interpretations focused on the topic word. The shifted visual attention to topic words supports the transformational account of emergent interpretations, in that the topic words were gazed at longer when the *ad hoc* category information for the vehicle characteristics was transformed to topic-related information. Previous neuroimaging studies have suggested that searching for a wider range of semantic relationships within metaphor comprehension elicits activation of the right inferior frontal gyrus (Stringaris et al., 2006; Shibata et al., 2007a). Greater activations were observed in the right precentral gyrus and anterior cingulate when reading novel metaphors compared to literal sentences. Moreover, the right precentral activation was greater compared to when reading familiar metaphors, which suggest that the involvement of precentral was specific to reading novel metaphors (Ahrens et al., 2007). Collectively, such neuroimaging evidence might suggest a right hemisphere contribution in metaphor comprehension, which contrasts with conventional knowledge about left-hemisphere dominant language processing. It is suggested that the application of neuroimaging methods to the current task might allow an examination of dynamic aspects (e.g., Jimura and Braver, 2010; Jimura et al., 2010, 2013) of this hypothesis.

In the present study, emergent interpretations were generated more frequently in the metaphor sentence conditions than in the literal condition. Moreover, the emergent interpretations were more unrelated to the topic and vehicle words for the novel metaphor than for the familiar metaphors and the literal sentences. These results suggest that novel metaphors, where the degree of dissimilarity is greater between the topic and the vehicle, yields interpretations that are more creative in terms of both their quantity and their quality. On the other hand, within prior studies, it has been suggested that one important factor of creative insight is the distinctiveness of the two concepts blended when insight occurs (Nagai et al., 2009).

Attentional shifts toward the topic words were observed about 8 s prior to the generation of emergent interpretations. It is unlikely that visual attention directly reflects semantic processing of the vehicle and topic words, such as semantic attention, formation of *ad hoc* categories, or combining of the distinct concepts. Nonetheless, it is not unreasonable to assume that the enhanced visual attention reflects some cognitive processes specifically associated with the generation of creative interpretations, such as semantic encoding (Huettig and Altmann, 2005), or representational changes (Knoblich et al., 2001).

The present study has quantitatively identified a period that may be important for creative insights. At the same time, the present study arises one critical question: what is occurring during the period starting with the attentional shift and ending when a response has been generated? One possible approach to finding an answer would be to monitor brain activity during this period, and to examine the functional dynamics that bring about the creative thoughts.

## Conflict of Interest Statement

The authors declare that the research was conducted in the absence of any commercial or financial relationships that could be construed as a potential conflict of interest.

## Footnotes

1 An illustrative example is that the metaphor “A child is a sponge” can be interpreted as “releases”, but “releases” is not a typical characteristic of either “child” or “sponge” (Becker, 1997).
