# Supplementary material for: Enhancement of visual attention precedes the emergence of novel metaphor interpretations
Source: Front Psychol. 2015 Jun 26;6:892. doi: 10.3389/fpsyg.2015.00892 (PMC4481861; doi:10.3389/fpsyg.2015.00892)
Supplement: Supplementary file 1 [file Table_1.PDF]

Supplementary Material

Enhancement of visual attention precedes the emergence of novel metaphor interpretations

Asuka Terai[1*], Masanori Nakagawa[1], Takashi Kusumi[2], Yasuharu Koike[3], Koji Jimura[4*]

[1] Department of Human System Science, Graduate School of Decision Science and Technology, Tokyo Institute of Technology, Tokyo, JAPAN
[2] Division of Cognitive Psychology in Education, Graduate School of Education, Kyoto University, Kyoto, JAPAN
[3] Precision & Intelligence Laboratory, Tokyo Institute of Technology, Tokyo, JAPAN
[4] Department of Biosciences and Informatics, Keio University, Tokyo, JAPAN

*** Correspondence:**
Asuka Terai, PhD
Department of Human System Science
Tokyo Institute of Technology
2-12-1 Oookayama
Meguroku, Tokyo, 152-8550, Japan
Email: terai.a.aa@m.titech.ac.jp

Koji Jimura, PhD
Department of Biosciences and Informatics
Keio University
3-14-1, Hiyoshi
Kohoku-ku Yokohama, 223-0061, Japan
Email: jimura@bio.keio.ac.jp

1. Supplementary Data

Ninety sentences (30 Novel metaphors, 30 Familiar metaphors, and 30 Literal sentences) were prepared and the sentences were divided into two sets with sentence conditions (N, F, L) matched within each set. Each participant was presented one of the two sentence sets. The original sentences were in Japanese and their translation is shown in Table 1 below.

Supplementary Table 1. Sentence Lists

Set 1		
Novel metaphor	Familiar metaphor	Literal Sentence
Fatigue is a window	Fatigue is baggage	Fatigue is a physical condition
Liquor is a dinosaur	Liquor is a friend	Liquor is alcohol
A herd is a crystal	A herd is a river	A herd is a crowd
Passion is homework	Passion is magma	Passion is a feeling
Judgment is a flower bed	Judgment is a balance scale	Judgment is a decision
A tree is juice	A tree is an umbrella	A tree is a plant
A discussion is a walk	A discussion is an architecture	A discussion is a conversation
A temptation is a pace	A temptation is honey	A temptation is an allurement
A memory is a dragonfly	A memory is a warehouse	A memory is a recollection

Hair is a hotel	Hair is silk thread	Hair is body hair
A truth is a shampoo	A truth is a labyrinth	A truth is a fact
A wheat field is a key	A wheat field is the sea	A wheat field is a farmland
A meteor is physical strength	A meteor is a raindrop	A meteor is an astronomical object
Love is a pair of slippers	Love is a season	Love is a concept
A fog is a chestnut	A fog is a mantle	A fog is moisture

Set 2		
Novel metaphor	Familiar metaphor	Literal Sentence
A room is a ball game	A room is a bottom of the sea	A room is a space
Marriage is a hat	Marriage is a refrigerator	Marriage is a contract
Education is an eel	Education is stairs	Education is learning
A kitten is a cool summer	A kitten is a princess	A kitten is a mammal
A revolution is a postal service	A revolution is an earthquake	A revolution is a reformation
Smile is a vase	Smile is ripples	Smile is an expression
Knowledge is a corridor	Knowledge is food	Knowledge is information
Authority is a stomach	Authority is a drug	Authority is domination
A heart is vegetable	A heart is a clock	A heart is an organ
Anxiety is an apple	Anxiety is a thick fog	Anxiety is a condition
Music is swimming	Music is medicine	Music is an art
A cigarette is a fish	A cigarette is a bomb	A cigarette is a product
A hope is a rule	A hope is a light	A hope is a wish
A dictionary is noodles	A dictionary is a gold mine	A dictionary is a book
Animosity is a cosmos	Animosity is poison	Animosity is an emotion
